# Guest Editorial

**Published:** 2015-08-11

**Authors:** HV Kambalimath

**Affiliations:** Secretary, Indian Society of Pedodontics and Preventive Dentistry, Professor and Head, Department of Pedodontics and Preventive Dentistry, Rishi Raj College of Dental Sciences and Research Centre Bhopal, Madhya Pradesh, India

Delay is the deadliest form of denial

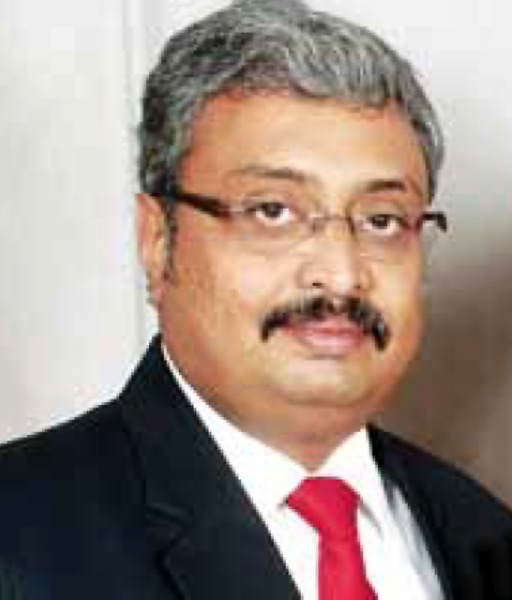

C Northcote Parkinson

In medical dictionaries, the word periodontium comes from the Greek terms ‘peri’, which means around, and ‘odons’, which means tooth. Literally, it means the part which is around the tooth. Periodontium includes the tissues that surround and support the teeth. Those tissues are gingiva, cementum, periodontal ligaments, and alveolar bone. It was found long ago that periodontium of the primary dentition differs from that of the permanent one; therefore, the protocol for early detection, prevention and management of periodontal conditions needs to be looked upon differently.

Periodontal diseases constitute a group of conditions that are nowadays considered to be ubiquitous among children, adolescents and adults. The term ‘periodontal diseases’ include any inherited or acquired disorders of the tissues that are investing and supporting the teeth.

Epidemiologic surveys with a widely varied background have been performed in many parts of the world among young individuals indicating gingivitis of varying severity. Normal and abnormal fluctuation in hormone levels, including changes in gonadotrophic hormone levels during the onset of puberty, can modify the gingival inflammatory response to dental plaque. Chronic gingivitis is the most commonly found clinical condition in children and it usually responds to thorough removal of bacterial deposits and improved daily oral hygiene. However, it is the most common condition found in children. Among periodontitis, aggressive periodontitis may be more common in children as compared to adults. Whenever periodontitis is present in children, it reflects some systemic abnormality that might exist because almost every time, periodontitis is a secondary manifestation of a systemic disease. So, it acts as mirror of systemic condition and early diagnosis can lead to decreased severity, improved prognosis of underlying disease.

The general dental practitioners and pediatric dentists are in a unique position to identify and distinguish between a seemingly innocuous condition that may be a normal physiological aberration or an early sign of severe destructive periodontal disease. Although severe destructive periodontal conditions are uncommon in children, it is essential that children receive a periodontal screening as part of their regular dental examination. Early diagnosis ensures a high likelihood of a successful therapeutic outcome, primarily by reduction of etiologic factors, remedial therapy and development of an effective maintenance protocol. This prevents the recurrence and progression of disease and reduces the incidence of tooth loss.

**HV Kambalimath**

Secretary, Indian Society of Pedodontics and Preventive Dentistry

Professor and Head

Department of Pedodontics and Preventive Dentistry

Rishi Raj College of Dental Sciences and Research Centre

Bhopal, Madhya Pradesh, India

